# Bis[1,3-bis­(2-cyano­phen­yl)triazenido]mercury(II)

**DOI:** 10.1107/S1600536809035326

**Published:** 2009-09-09

**Authors:** Mohammad Kazem Rofouei, Marzieh Barghamadi, Mehdi Taghdiri, Jafar Attar Gharamaleki

**Affiliations:** a Faculty of Chemistry, Tarbiat Moallem University, Tehran, Iran; b Department of Chemistry, Payame Noor University, Ardakan, Yazd, Iran; c Young Researchers Club, Islamic Azad University, North Tehran Branch, Tehran, Iran

## Abstract

In the title compound, [Hg(C_14_H_8_N_5_)_2_], the central atom is four-coordinated by two bidentate 1,3-bis­(2-cyano­phen­yl)triazenide ligands in a distorted square-planar geometry. The asymmteric unit is composed of one ligand molecule and one Hg^II^ ion, which is disordered over two sites, one lying on an inversion center and the other on a general position with site-occupancy factors of 0.2378 (7) and 0.3811 (7), respectively. The monomeric mol­ecules of the complex are linked into pairs through non-classical C—H⋯N hydrogen bonds. The resulting dimeric units are assembled by translation along the crystallographic *c* axis into chains linked through secondary π–π inter­actions [centroid–centroid distances = 3.685 (2) and 3.574 (2) Å], as well as C—H⋯π stacking inter­actions, resulting in a two-dimensional architecture.

## Related literature

For transition metal complexes containing 1,3-diaryl­triazenide ions, see: Vrieze & Van Koten (1987 ▶); Hursthouse *et al.* (1993 ▶). For metal–η-arene π-inter­actions in Hg^II^ complexes, see: Horner *et al.* (2006 ▶). For related crystal structures, see: Rofouei *et al.* (2006 ▶); Melardi *et al.* (2008 ▶); Payehghadr *et al.* (2006 ▶); Melardi *et al.* (2007 ▶); Hematyar & Rofouei (2008 ▶); Rofouei *et al.* (2009 ▶).
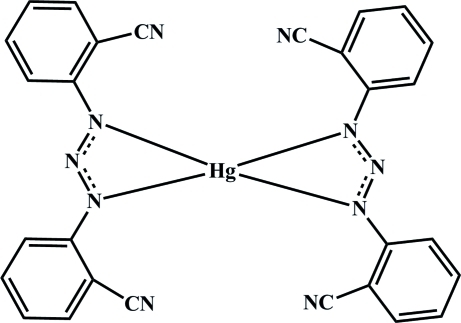


## Experimental

### 

#### Crystal data

[Hg(C_14_H_8_N_5_)_2_]
*M* *_r_* = 693.50
Monoclinic, 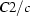

*a* = 23.0721 (12) Å
*b* = 7.7307 (4) Å
*c* = 15.6680 (8) Å
β = 110.481 (1)°
*V* = 2617.9 (2) Å^3^
*Z* = 4
Mo *K*α radiation
μ = 5.92 mm^−1^
*T* = 100 K
0.21 × 0.20 × 0.08 mm

#### Data collection

Bruker APEXII CCD area-detector diffractometer
Absorption correction: multi-scan (*SADABS*; Bruker, 2005 ▶) *T*_min_ = 0.296, *T*_max_ = 0.629
15538 measured reflections
3479 independent reflections
2876 reflections with *I* > 2σ(*I*)
*R*_int_ = 0.044

#### Refinement

*R*[*F* ^2^ > 2σ(*F* ^2^)] = 0.034
*wR*(*F* ^2^) = 0.085
*S* = 0.99
3479 reflections
183 parameters
H-atom parameters constrained
Δρ_max_ = 2.67 e Å^−3^
Δρ_min_ = −1.12 e Å^−3^

### 

Data collection: *APEX2* (Bruker, 2005 ▶); cell refinement: *SAINT* (Bruker, 2005 ▶); data reduction: *SAINT*; program(s) used to solve structure: *SHELXS97* (Sheldrick, 2008 ▶); program(s) used to refine structure: *SHELXL97* (Sheldrick, 2008 ▶); molecular graphics: *SHELXTL* (Sheldrick, 2008 ▶); software used to prepare material for publication: *SHELXTL*.

## Supplementary Material

Crystal structure: contains datablocks I, global. DOI: 10.1107/S1600536809035326/pv2202sup1.cif

Structure factors: contains datablocks I. DOI: 10.1107/S1600536809035326/pv2202Isup2.hkl

Additional supplementary materials:  crystallographic information; 3D view; checkCIF report

## Figures and Tables

**Table 1:** Hydrogen-bond geometry (Å, °)

*D*—H⋯*A*	*D*—H	H⋯*A*	*D*⋯*A*	*D*—H⋯*A*
C4—H4*A*⋯N4^i^	0.95	2.61	3.428 (5)	145
C6—H6*A*⋯N5^ii^	0.95	2.61	3.522 (5)	160
C10—H10*A*⋯*Cg*2^iii^	0.95	2.96	3.629 (2)	129

Symmetry codes: (i) 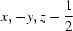; (ii) 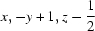; (iii) 
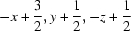
. *Cg2* is the centroid of C7–C12 ring.

**Fig. 1.**
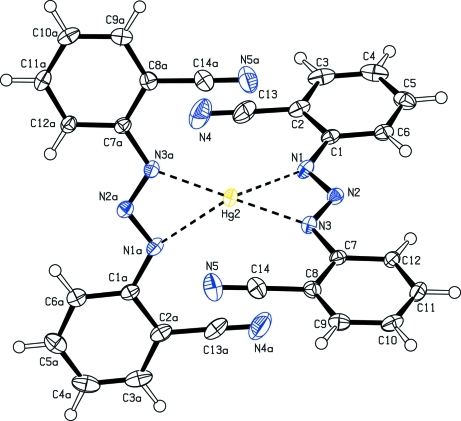
Molecular structure of the title compound, with ellipsoids drawn at 50% probability level: Hg1 has been omitted for clarity. Symmetry code to generate atoms with label "a": -x + 1, y, -z + 1/2.

**Fig. 2.**
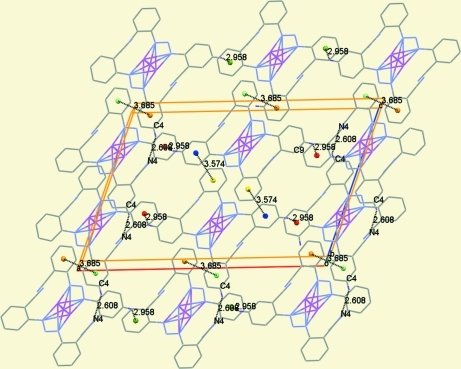
The unit cell packing diagram of the title compound along b crystal axes showing π-π and C–H···π stacking interactions. Hydrogen atoms have been excluded for clarity.

## supplementary crystallographic information

### Comment

Triazenes are characterized by a diazoamino group (NN—N) commonly adopting a *trans* configuration in the ground state. Recently, the study of transition metal complexes containing 1,3-diaryltriazenide ions has been increased due to the potential reactivity of these ligands (Vrieze & Koten, 1987). This anion is a three-atom donor ligand that can act as a monodentate group, a chelating ligand or a bridging ligand between two metal centers (Hursthouse *et al.*, 1993).

Several bisdiaryl symmetric and asymmetric-substituted triazenide complex polymers of Hg(II) which have a remarkable ability to self-assemble in different manners through metal-η-arene π-interactions have been reported (Horner *et al.*, 20006). Hg(II) is a diamagnetic ion and maintains d^10^ electron configuration which minimizes intrinsic coordination geometry preferences while favoring coordination by softer ligands. We have previously reported the synthesis of [1,3-bis(2-methoxy)phenyl]triazene ligand (Rofouei *et al.*, 2006) and its Ag(I) complex (Payehghadr *et al.*, 2006). In addition, we have reported the Hg(II) complexes with this ligand by using HgCl_2_ (Melardi *et al.*, 2007), HgBr_2_ (Hematyar and Rofouei, 2008), Hg(CH_3_COO)_2_ and Hg(SCN)_2_ (Rofouei *et al.*, 2009) as starting materials. In this paper, a new Hg(II) complex of a recently synthesized ligand ([1,3-bis(2-cyano)phenyl]triazene) (Melardi *et al.*, 2008) is reported. Mercury(II) acetate was used as the starting material.

The molecular structure of the title compound is shown in Fig. 1.  There is a high disorder of Hg atom in the complex. The Hg(II) atom was disordered over 3 sites (only 2 of them are symmetrically independent). Hg1:Hg1A:Hg2 atoms are disordered in ratio 0.38:0.38:0.24. The central atom in this compound is coordinated by two symmetrically related triazenide ions. Each Hg^II^ atom is four-coordinated by two nitrogen atoms N1, and N3 of two 1,3-bis(2-cyano)phenyl]triazene fragments which act as bidentate ligands. The arrangement of the four donor atoms around Hg(II) atom results in a distorted square planar geometry.

In the lattice of the title compound, the monomeric [Hg(C_14_H_8_N_5_)_2_] molecules are linked into pairs through non-classical C–H···N hydrogen bonds (details are provided in Table 1). The resulting dimeric units are assembled by translation along the crystallographic *c *axis to unidimensional chains linked through secondary π–interactions. Consequently, 1-D chains formed by C–H···N hydrogen bonds are connected with one another by π-π and C–H···π stacking interactions, resulting in the 2-D architecture(Fig. 2). These π-π stacking interactions are present between aromatic rings with centroid-centroid distances of 3.685 (2) and 3.574 (2) Å and also between C–H group of phenyl rings with aromatic rings with H···π distance of 2.96 Å for C10—H10A···*Cg*; *Cg* is the centroid of C7—C12 ring.

### Experimental

Anhydrous methanolic solution of [1,3-bis(2-cyano)phenyl]triazene (0.247 g) was added to anhydrous methanolic solution of mercury(II) acetate ( 0.160 g). After several hours, the mixture was filtered, the product washed with methanol, the yellow precipitate thus obtained was dissolved in diethyl ether and stored in a freezer. After 14 days, well formed orange-red crystals were produced which decompose above 523 K.

### Refinement

There is a high disorder of Hg atom in the comples. The Hg atom in the complex was disordered over 3 sites (only 2 of them are symmetrically independent), where disorder was refined as free variable (using FVAR instrcution).

Positions of H-atoms were calculated and refined in isotropic approximation in riding mode with (C–H = 0.95 Å) and *U*_iso_(H) = 1.2*U*_eq_(parent C).

There is a high positive residual density of 2.67 e Å^-3^ near the Hg1 center (distance 0.85% A) due to considerable absorption effects which could not be completely corrected.

### Crystal data

**Table:**

[Hg(C_14_H_8_N_5_)_2_]	*F*(000) = 1336
*M**_r_* = 693.50	*D*_x_ = 1.759 Mg m^−^^3^
Monoclinic, *C*2/*c*	Mo *K*α radiation, λ = 0.71073 Å
Hall symbol: -C 2yc	Cell parameters from 3560 reflections
*a* = 23.0721 (12) Å	θ = 2.8–30.1°
*b* = 7.7307 (4) Å	µ = 5.92 mm^−^^1^
*c* = 15.6680 (8) Å	*T* = 100 K
β = 110.481 (1)°	Plate, orange
*V* = 2617.9 (2) Å^3^	0.21 × 0.20 × 0.08 mm
*Z* = 4	

### Data collection

**Table:**

Bruker APEXII CCD area-detector diffractometer	3479 independent reflections
Radiation source: fine-focus sealed tube	2876 reflections with *I* > 2σ(*I*)
graphite	*R*_int_ = 0.044
ω scans	θ_max_ = 29.0°, θ_min_ = 1.9°
Absorption correction: multi-scan (*SADABS*; Bruker, 2005)	*h* = −31→31
*T*_min_ = 0.296, *T*_max_ = 0.629	*k* = −10→10
15538 measured reflections	*l* = −21→21

### Refinement

**Table:**

Refinement on *F*^2^	Primary atom site location: structure-invariant direct methods
Least-squares matrix: full	Secondary atom site location: difference Fourier map
*R*[*F*^2^ > 2σ(*F*^2^)] = 0.034	Hydrogen site location: inferred from neighbouring sites
*wR*(*F*^2^) = 0.085	H-atom parameters constrained
*S* = 0.99	*w* = 1/[σ^2^(*F*_o_^2^) + (0.04*P*)^2^ + 7*P*] where *P* = (*F*_o_^2^ + 2*F*_c_^2^)/3
3479 reflections	(Δ/σ)_max_ = 0.001
183 parameters	Δρ_max_ = 2.67 e Å^−^^3^
0 restraints	Δρ_min_ = −1.12 e Å^−^^3^

### Special details

**Table:**

Experimental. ^1^H NMR and ^13^C NMR of the free ligand and the title compound were recorded in DMSO as solvent:For the free ligand, hydrogen atoms of the aromatic ring appear at δ = 7.27–7.86 ppm (4H) and hydrogen atom of NH group appears at δ = 13.45 ppm (1H); in ^13^C NMR we have resonances at 118, 124, 128, 133, 134 and 135 ppm which belong to the carbon atoms of aromatic ring. In ^1^H NMR spectra of the title complex, the resonance for the hydrogen atom of NH group has completely disappeared and the hydrogen atoms of aromatic ring now appear at δ = 7.27–7.86 ppm (4H); in ^13^C NMR spectra, carbon atoms of aromatic ring have resonances at 103, 118, 120, 125, 133 and 149 ppm. These data support the structure presented in this article.
Geometry. All e.s.d.'s (except the e.s.d. in the dihedral angle between two l.s. planes) are estimated using the full covariance matrix. The cell e.s.d.'s are taken into account individually in the estimation of e.s.d.'s in distances, angles and torsion angles; correlations between e.s.d.'s in cell parameters are only used when they are defined by crystal symmetry. An approximate (isotropic) treatment of cell e.s.d.'s is used for estimating e.s.d.'s involving l.s. planes.
Refinement. Refinement of *F*^2^ against ALL reflections. The weighted *R*-factor *wR* and goodness of fit *S* are based on *F*^2^, conventional *R*-factors *R* are based on *F*, with *F* set to zero for negative *F*^2^. The threshold expression of *F*^2^ > σ(*F*^2^) is used only for calculating *R*-factors(gt) *etc*. and is not relevant to the choice of reflections for refinement. *R*-factors based on *F*^2^ are statistically about twice as large as those based on *F*, and *R*- factors based on ALL data will be even larger.

### Fractional atomic coordinates and isotropic or equivalent isotropic displacement parameters (Å^2^)

**Table:**

	*x*	*y*	*z*	*U*_iso_*/*U*_eq_	Occ. (<1)
Hg1	0.467164 (12)	0.37562 (3)	0.213648 (18)	0.01852 (8)	0.3811 (7)
Hg2	0.5000	0.44451 (8)	0.2500	0.01852 (8)	0.2378 (7)
C1	0.46005 (11)	0.2107 (3)	0.02350 (18)	0.0190 (5)	
C2	0.40569 (13)	0.1222 (4)	0.0192 (2)	0.0256 (6)	
C3	0.37257 (13)	0.0268 (4)	−0.0586 (2)	0.0313 (7)	
H3A	0.3360	−0.0328	−0.0611	0.038*	
C4	0.39267 (15)	0.0190 (4)	−0.1315 (2)	0.0337 (7)	
H4A	0.3703	−0.0458	−0.1842	0.040*	
C5	0.44625 (14)	0.1073 (4)	−0.1270 (2)	0.0288 (6)	
H5A	0.4600	0.1022	−0.1774	0.035*	
C6	0.48006 (12)	0.2024 (4)	−0.05072 (18)	0.0215 (5)	
H6A	0.5165	0.2615	−0.0491	0.026*	
C7	0.62706 (12)	0.5268 (3)	0.19697 (18)	0.0183 (5)	
C8	0.65811 (13)	0.6146 (4)	0.27876 (19)	0.0216 (5)	
C9	0.71486 (13)	0.6961 (4)	0.2931 (2)	0.0256 (6)	
H9A	0.7353	0.7553	0.3487	0.031*	
C10	0.74111 (13)	0.6907 (4)	0.2268 (2)	0.0269 (6)	
H10A	0.7797	0.7459	0.2364	0.032*	
C11	0.71085 (13)	0.6038 (4)	0.1457 (2)	0.0244 (6)	
H11A	0.7290	0.6007	0.0999	0.029*	
C12	0.65480 (12)	0.5218 (4)	0.13037 (18)	0.0199 (5)	
H12A	0.6351	0.4620	0.0747	0.024*	
C13	0.38358 (14)	0.1295 (4)	0.0939 (3)	0.0359 (8)	
C14	0.63072 (15)	0.6272 (4)	0.3485 (2)	0.0287 (6)	
N1	0.49222 (10)	0.2999 (3)	0.10491 (16)	0.0209 (5)	
N2	0.54323 (10)	0.3720 (3)	0.10789 (15)	0.0192 (5)	
N3	0.56967 (10)	0.4507 (3)	0.18522 (15)	0.0189 (5)	
N4	0.36490 (15)	0.1358 (5)	0.1528 (3)	0.0531 (9)	
N5	0.60964 (15)	0.6392 (4)	0.4044 (2)	0.0416 (7)	

### Atomic displacement parameters (Å^2^)

**Table:**

	*U*^11^	*U*^22^	*U*^33^	*U*^12^	*U*^13^	*U*^23^
Hg1	0.01873 (13)	0.01983 (13)	0.02026 (13)	−0.00001 (10)	0.01092 (9)	−0.00188 (10)
Hg2	0.01873 (13)	0.01983 (13)	0.02026 (13)	−0.00001 (10)	0.01092 (9)	−0.00188 (10)
C1	0.0150 (12)	0.0155 (12)	0.0251 (13)	0.0019 (10)	0.0053 (10)	0.0025 (10)
C2	0.0172 (13)	0.0223 (14)	0.0358 (16)	0.0018 (11)	0.0074 (11)	0.0029 (12)
C3	0.0189 (14)	0.0213 (15)	0.0452 (18)	−0.0002 (11)	0.0005 (13)	0.0025 (13)
C4	0.0314 (16)	0.0237 (15)	0.0330 (17)	0.0033 (12)	−0.0052 (13)	−0.0015 (12)
C5	0.0332 (16)	0.0246 (15)	0.0239 (14)	0.0079 (12)	0.0040 (12)	0.0015 (11)
C6	0.0206 (13)	0.0160 (12)	0.0265 (14)	0.0048 (10)	0.0065 (11)	0.0036 (11)
C7	0.0154 (12)	0.0180 (12)	0.0210 (12)	0.0014 (9)	0.0058 (10)	0.0033 (10)
C8	0.0237 (13)	0.0199 (13)	0.0208 (13)	0.0030 (10)	0.0073 (11)	0.0030 (10)
C9	0.0232 (14)	0.0235 (14)	0.0252 (14)	−0.0004 (11)	0.0022 (11)	−0.0012 (11)
C10	0.0156 (13)	0.0256 (14)	0.0374 (16)	0.0002 (11)	0.0065 (12)	0.0041 (13)
C11	0.0217 (13)	0.0246 (15)	0.0316 (15)	0.0025 (11)	0.0152 (12)	0.0040 (11)
C12	0.0169 (12)	0.0222 (14)	0.0216 (13)	0.0020 (10)	0.0080 (10)	0.0029 (10)
C13	0.0229 (15)	0.0358 (18)	0.052 (2)	−0.0071 (13)	0.0167 (15)	0.0026 (15)
C14	0.0357 (16)	0.0265 (15)	0.0241 (14)	−0.0007 (13)	0.0106 (13)	−0.0005 (12)
N1	0.0177 (11)	0.0189 (11)	0.0291 (12)	−0.0018 (9)	0.0120 (9)	−0.0014 (9)
N2	0.0177 (10)	0.0186 (11)	0.0220 (11)	0.0002 (9)	0.0080 (9)	0.0024 (9)
N3	0.0193 (11)	0.0187 (11)	0.0209 (11)	−0.0009 (9)	0.0098 (9)	−0.0006 (9)
N4	0.0406 (18)	0.064 (2)	0.067 (2)	−0.0130 (16)	0.0348 (17)	−0.0032 (18)
N5	0.0542 (19)	0.0458 (18)	0.0313 (15)	−0.0025 (14)	0.0231 (14)	−0.0045 (13)

### Geometric parameters (Å, °)

**Table:**

Hg1—Hg2	0.9381 (4)	C5—H5A	0.9500
Hg1—Hg1^i^	1.5445 (5)	C6—H6A	0.9500
Hg1—N1	2.066 (2)	C7—N3	1.401 (3)
Hg1—N3^i^	2.124 (2)	C7—C12	1.403 (4)
Hg1—N3	2.620 (2)	C7—C8	1.405 (4)
Hg2—Hg1^i^	0.9381 (4)	C8—C9	1.399 (4)
Hg2—N3	2.181 (2)	C8—C14	1.444 (4)
Hg2—N3^i^	2.181 (2)	C9—C10	1.374 (4)
Hg2—N1	2.483 (2)	C9—H9A	0.9500
Hg2—N1^i^	2.483 (2)	C10—C11	1.390 (4)
C1—C6	1.395 (4)	C10—H10A	0.9500
C1—C2	1.409 (4)	C11—C12	1.384 (4)
C1—N1	1.411 (3)	C11—H11A	0.9500
C2—C3	1.401 (4)	C12—H12A	0.9500
C2—C13	1.432 (5)	C13—N4	1.148 (5)
C3—C4	1.376 (5)	C14—N5	1.145 (4)
C3—H3A	0.9500	N1—N2	1.288 (3)
C4—C5	1.392 (5)	N2—N3	1.301 (3)
C4—H4A	0.9500	N3—Hg1^i^	2.124 (2)
C5—C6	1.388 (4)		
			
N1—Hg1—N3^i^	173.18 (9)	C9—C8—C7	121.0 (3)
N1—Hg1—N3	52.63 (8)	C9—C8—C14	118.7 (3)
N3^i^—Hg1—N3	133.38 (8)	C7—C8—C14	120.3 (3)
N3—Hg2—N3^i^	177.49 (12)	C10—C9—C8	120.0 (3)
N3—Hg2—N1	54.04 (8)	C10—C9—H9A	120.0
N3^i^—Hg2—N1	127.37 (8)	C8—C9—H9A	120.0
N3—Hg2—N1^i^	127.37 (8)	C9—C10—C11	119.5 (3)
N3^i^—Hg2—N1^i^	54.04 (8)	C9—C10—H10A	120.2
N1—Hg2—N1^i^	126.46 (11)	C11—C10—H10A	120.2
C6—C1—C2	119.1 (3)	C12—C11—C10	121.4 (3)
C6—C1—N1	123.6 (2)	C12—C11—H11A	119.3
C2—C1—N1	117.3 (2)	C10—C11—H11A	119.3
C3—C2—C1	120.1 (3)	C11—C12—C7	120.0 (3)
C3—C2—C13	119.3 (3)	C11—C12—H12A	120.0
C1—C2—C13	120.5 (3)	C7—C12—H12A	120.0
C4—C3—C2	120.4 (3)	N4—C13—C2	178.9 (4)
C4—C3—H3A	119.8	N5—C14—C8	178.9 (4)
C2—C3—H3A	119.8	N2—N1—C1	115.4 (2)
C3—C4—C5	119.2 (3)	N2—N1—Hg1	111.29 (18)
C3—C4—H4A	120.4	C1—N1—Hg1	132.34 (17)
C5—C4—H4A	120.4	N2—N1—Hg2	89.96 (16)
C6—C5—C4	121.6 (3)	C1—N1—Hg2	153.44 (17)
C6—C5—H5A	119.2	N1—N2—N3	111.2 (2)
C4—C5—H5A	119.2	N2—N3—C7	115.6 (2)
C5—C6—C1	119.5 (3)	N2—N3—Hg1^i^	112.79 (16)
C5—C6—H6A	120.3	C7—N3—Hg1^i^	128.55 (17)
C1—C6—H6A	120.3	N2—N3—Hg2	103.97 (16)
N3—C7—C12	123.1 (2)	C7—N3—Hg2	139.87 (18)
N3—C7—C8	118.7 (2)	N2—N3—Hg1	84.13 (14)
C12—C7—C8	118.2 (2)	C7—N3—Hg1	159.80 (18)
			
N1—Hg1—Hg2—Hg1^i^	−80.24 (7)	N3^i^—Hg2—N1—N2	171.78 (14)
N3^i^—Hg1—Hg2—Hg1^i^	103.00 (6)	N1^i^—Hg2—N1—N2	−118.98 (15)
N3—Hg1—Hg2—Hg1^i^	−79.11 (7)	Hg1—Hg2—N1—C1	12.3 (4)
Hg1^i^—Hg1—Hg2—N3	79.11 (7)	Hg1^i^—Hg2—N1—C1	124.7 (4)
N1—Hg1—Hg2—N3	−1.13 (10)	N3—Hg2—N1—C1	−169.1 (4)
N3^i^—Hg1—Hg2—N3	−177.89 (11)	N3^i^—Hg2—N1—C1	8.4 (4)
Hg1^i^—Hg1—Hg2—N3^i^	−103.00 (6)	N1^i^—Hg2—N1—C1	77.6 (4)
N1—Hg1—Hg2—N3^i^	176.76 (9)	Hg1^i^—Hg2—N1—Hg1	112.41 (8)
N3—Hg1—Hg2—N3^i^	177.89 (11)	N3—Hg2—N1—Hg1	178.67 (12)
Hg1^i^—Hg1—Hg2—N1	80.24 (7)	N3^i^—Hg2—N1—Hg1	−3.92 (11)
N3^i^—Hg1—Hg2—N1	−176.76 (9)	N1^i^—Hg2—N1—Hg1	65.32 (5)
N3—Hg1—Hg2—N1	1.13 (10)	C1—N1—N2—N3	−179.9 (2)
Hg1^i^—Hg1—Hg2—N1^i^	−52.59 (5)	Hg1—N1—N2—N3	9.9 (3)
N1—Hg1—Hg2—N1^i^	−132.83 (10)	Hg2—N1—N2—N3	8.2 (2)
N3^i^—Hg1—Hg2—N1^i^	50.42 (8)	N1—N2—N3—C7	177.2 (2)
N3—Hg1—Hg2—N1^i^	−131.70 (9)	N1—N2—N3—Hg1^i^	15.2 (3)
C6—C1—C2—C3	0.3 (4)	N1—N2—N3—Hg2	−9.6 (2)
N1—C1—C2—C3	−178.1 (2)	N1—N2—N3—Hg1	−7.29 (19)
C6—C1—C2—C13	−179.4 (3)	C12—C7—N3—N2	0.6 (4)
N1—C1—C2—C13	2.2 (4)	C8—C7—N3—N2	179.8 (2)
C1—C2—C3—C4	−0.2 (4)	C12—C7—N3—Hg1^i^	159.3 (2)
C13—C2—C3—C4	179.5 (3)	C8—C7—N3—Hg1^i^	−21.6 (4)
C2—C3—C4—C5	−0.1 (4)	C12—C7—N3—Hg2	−169.1 (2)
C3—C4—C5—C6	0.3 (4)	C8—C7—N3—Hg2	10.1 (4)
C4—C5—C6—C1	−0.1 (4)	C12—C7—N3—Hg1	−166.4 (4)
C2—C1—C6—C5	−0.1 (4)	C8—C7—N3—Hg1	12.7 (7)
N1—C1—C6—C5	178.1 (2)	Hg1—Hg2—N3—N2	6.87 (18)
N3—C7—C8—C9	−178.6 (2)	Hg1^i^—Hg2—N3—N2	114.18 (16)
C12—C7—C8—C9	0.6 (4)	N1—Hg2—N3—N2	5.75 (14)
N3—C7—C8—C14	−0.7 (4)	N1^i^—Hg2—N3—N2	117.46 (16)
C12—C7—C8—C14	178.4 (3)	Hg1—Hg2—N3—C7	177.3 (3)
C7—C8—C9—C10	−0.2 (4)	Hg1^i^—Hg2—N3—C7	−75.4 (3)
C14—C8—C9—C10	−178.1 (3)	N1—Hg2—N3—C7	176.2 (3)
C8—C9—C10—C11	0.0 (4)	N1^i^—Hg2—N3—C7	−72.1 (3)
C9—C10—C11—C12	−0.3 (4)	N1—Hg2—N3—Hg1^i^	−108.43 (9)
C10—C11—C12—C7	0.7 (4)	N1^i^—Hg2—N3—Hg1^i^	3.28 (9)
N3—C7—C12—C11	178.3 (2)	Hg1^i^—Hg2—N3—Hg1	107.31 (8)
C8—C7—C12—C11	−0.8 (4)	N1—Hg2—N3—Hg1	−1.12 (10)
C6—C1—N1—N2	−1.5 (4)	N1^i^—Hg2—N3—Hg1	110.59 (11)
C2—C1—N1—N2	176.7 (2)	Hg2—Hg1—N3—N2	−173.30 (17)
C6—C1—N1—Hg1	166.1 (2)	Hg1^i^—Hg1—N3—N2	143.26 (16)
C2—C1—N1—Hg1	−15.7 (4)	N1—Hg1—N3—N2	5.33 (14)
C6—C1—N1—Hg2	160.0 (3)	N3^i^—Hg1—N3—N2	−170.43 (12)
C2—C1—N1—Hg2	−21.7 (5)	Hg2—Hg1—N3—C7	−5.0 (5)
Hg2—Hg1—N1—N2	−4.62 (19)	Hg1^i^—Hg1—N3—C7	−48.5 (5)
Hg1^i^—Hg1—N1—N2	−38.93 (18)	N1—Hg1—N3—C7	173.6 (5)
N3—Hg1—N1—N2	−5.75 (15)	N3^i^—Hg1—N3—C7	−2.2 (6)
Hg1^i^—Hg1—N1—C1	153.1 (2)	Hg2—Hg1—N3—Hg1^i^	43.45 (5)
N3—Hg1—N1—C1	−173.7 (3)	N1—Hg1—N3—Hg1^i^	−137.93 (11)
Hg1^i^—Hg1—N1—Hg2	−34.32 (4)	N3^i^—Hg1—N3—Hg1^i^	46.32 (14)
N3—Hg1—N1—Hg2	−1.13 (10)	Hg1^i^—Hg1—N3—Hg2	−43.45 (5)
Hg1—Hg2—N1—N2	175.70 (18)	N1—Hg1—N3—Hg2	178.63 (12)
Hg1^i^—Hg2—N1—N2	−71.89 (15)	N3^i^—Hg1—N3—Hg2	2.87 (14)
N3—Hg2—N1—N2	−5.63 (14)		

Symmetry codes: (i) −*x*+1, *y*, −*z*+1/2.

### Hydrogen-bond geometry (Å, °)

**Table:**

*D*—H···*A*	*D*—H	H···*A*	*D*···*A*	*D*—H···*A*
C4—H4A···N4^ii^	0.95	2.61	3.428 (5)	145
C6—H6A···N5^iii^	0.95	2.61	3.522 (5)	160
C10—H10A···Cg2^iv^	0.95	2.96	3.629 (2)	129

Symmetry codes: (ii) *x*, −*y*, *z*−1/2; (iii) *x*, −*y*+1, *z*−1/2; (iv) −*x*+3/2, *y*+1/2, −*z*+1/2.
